# Highly Self-Healable Polymeric Coating Materials Based on Charge Transfer Complex Interactions with Outstanding Weatherability

**DOI:** 10.3390/polym15234544

**Published:** 2023-11-27

**Authors:** Pyong Hwa Hong, Gyeongmin Moon, Jinsil Kim, Kiwon Choi, Min Jae Ko, Ho Gyu Yoon, Sung Woo Hong

**Affiliations:** 1 Green and Sustainable Materials R&D Department, Korea Institute of Industrial Technology, 89 Yangdaegiro-gil, Ipjang-myeon, Seobuk-gu, Cheonan-si 31056, Republic of Korea; 2 Department of Materials Science and Engineering, Korea University, 145 Anam-ro, Seongbuk-gu, Seoul 02841, Republic of Korea; 3 Department of Chemical Engineering, University of Montreal, 2900 Edouard Montpetit Blvd, Montreal, QC H3T 1J4, Canada; 4 Department of Chemical Engineering, Hanyang University, 222 Wangsimni-ro, Seongdong-gu, Seoul 04763, Republic of Korea; 5 Institute of Nano Science and Technology, Hanyang University, 222 Wangsimni-ro, Seongdong-gu, Seoul 04763, Republic of Korea; 6 Convergence Research Center for Solutions to Electromagnetic Interference in Future-Mobility, Korea Institute of Science and Technology, 5 Hwarang-ro 14-gil, Seongbuk-gu, Seoul 02792, Republic of Korea

**Keywords:** polymeric coating, self-healing, charge transfer complex (CTC), engineering plastic, weatherability

## Abstract

In this study, we prepare highly self-healable polymeric coating materials using charge transfer complex (CTC) interactions. The resulting coating materials demonstrate outstanding thermal stability (1 wt% loss thermal decomposition temperature at 420 °C), rapid self-healing kinetics (in 5 min), and high self-healing efficiency (over 99%), which is facilitated by CTC-induced multiple interactions between the polymeric chains. In addition, these materials exhibit excellent optical properties, including transmittance over 91% and yellow index (YI) below 2, and show enhanced weatherability with a ΔYI value below 0.5 after exposure to UV light for 72 h. Furthermore, the self-healable coating materials developed in this study show outstanding mechanical properties by overcoming the limitations of conventional self-healing materials.

## 1. Introduction

Polymer materials have exhibited significant potential for applications in next-generation electronics and mobilities. Compared with metals, ceramics, and carbon materials, they demonstrate numerous advantages, including lightweight, flexibility, ease of large-scale manufacturing, compatibility with various materials, and exceptional versatility, making them highly attractive [1]. Particularly, polymeric coating materials (PCMs) emerge as promising candidates for safeguarding the surfaces of products such as flexible displays, automobiles, medical equipment, and solar cells [2,3]. In addition, they play a crucial role in protecting the internal components below the surface against external stimuli, including impacts, scratches, moisture, high temperatures, chemical substances, acids, bases, and exposure to UV radiation [4,5].

PCMs can be used for flexible electronics owing to their important inherent characteristics, including flexibility, light weight, elasticity, and robust chemical durability [6]. However, they tend to compromise surface hardness compared with conventional hard coatings, making them susceptible to damage from impacts and scratches [7,8]. It is also noted that exposure to UV light has to be considered because it can generate radicals in the polymeric chain through photochemical reactions [9,10]. Consequently, these radicals lead to polymeric chain scission, which is a primary reason for reduced material durability and aesthetic degradation [11,12]. Therefore, PCMs must resist UV light regarding material lifespan [13,14]. Numerous researchers have carried out studies to address these issues using various strategies. These approaches involve incorporating functional groups to improve mechanical properties [15,16,17], enhancing surface hardness [18,19], integrating UV-stable components [20,21,22], and implementing self-healing moieties [23,24].

Self-healable PCMs have attracted significant attention because they can autonomously repair damages caused by external stimuli, thus extending the lifespan of surface coating materials [25,26,27]. Self-healing systems based on chemical and physical interactions can autonomously restore their damages and demonstrate a noteworthy capability for repetitive self-repair [28,29,30]. However, self-healing materials based on chemical interactions typically require elevated thermal energy, which can adversely affect the internal components of devices [31]. As a result, self-healing materials based on physical interactions emerge as a more suitable approach to protect the surface and internal components of the device. Typical physical interactions used for self-healing materials include ionic interaction [32,33,34], hydrogen interaction [35,36,37], host–guest interaction [38,39], metal–ligand coordination [40,41,42], and charge transfer complex interaction [43,44]. Because of the intrinsic reversibility of these interactions, self-healing materials based on physical interactions have the potential to efficiently and repeatedly repair damage at specific sites. However, conventional self-healable PCMs face challenges due to their low mechanical surface properties. This can lead to the loss of healable parts when they are subjected to significant damage, resulting in a drastic decrease in healing efficiency [26,45]. Therefore, strong mechanical surface properties that increase scratch resistance are crucial. Traditional self-healing materials often exhibit a trade-off between mechanical properties and self-healing characteristics [46,47]. These properties are closely associated with chain interactions and mobility [48]. The optimal balance between chain interactions and chain mobility improves the mechanical durability and self-healing ability of PCMs and plays an essential role in developing functional PCMs for next-generation electronics and mobilities.

To address the trade-off associated with conventional self-healing materials, engineering plastics-based PCMs emerge as promising candidates in coating applications. Engineering plastics are renowned for their exceptional mechanical properties and ability to withstand thermal, chemical, and environmental stimuli, distinguishing them from conventional polymers [49,50]. Polyimides (PIs), classified as representative engineering plastic, possess a unique internal structure known as a charge transfer complex (CTC) [51,52]. Because imide groups exhibit electron-withdrawing characteristics, an electron acceptor with a relative electron deficiency and an electron donor carrying abundant electrons are formed in the PI chain. This alternating electron donor–acceptor arrangement repeats along the PI chain, resulting in continuous interactions between electron donors and acceptors and the CTC formation [53,54]. Compared with other engineering plastics, PIs exhibit remarkable mechanical strength and thermal stability owing to CTC interactions [55,56]. It is noted that the high glass transition temperature (*T*_g_) of PI poses challenges when considering PI as a self-healing material. The high temperature required for chain mobility to initiate the self-healing process can trigger thermal degradation and property deterioration [57]. When employing PIs as PCMs, it is essential to maintain their outstanding mechanical and thermal properties while also achieving self-healing characteristics. Consequently, it has become imperative to effectively overcome the trade-off in engineering plastics-based PCMs and optimize the balance between their mechanical properties and self-healing capability.

In this study, we present functional PCMs based on CTC interactions with outstanding thermal properties, mechanical surface strength, self-healing efficiency, and weatherability. The mechanical properties and self-healing ability are investigated in terms of CTC interaction. Consequently, the self-healable PCMs effectively overcome the trade-off in self-healing materials while displaying improved mechanical surface properties and effective self-healing capabilities. Additionally, the optical properties and weatherability are characterized in terms of the chemical structure of the PI chain. We also propose a mechanism for improved self-healing properties of CTC-based PCMs developed in this study.

## 2. Materials and Methods

### 2.1. Materials

4,4′-(hexafluoroisopropylidene)diphthalic anhydride (6FDA) (Changzhou Sunlight Pharmaceutical Co., Ltd., Changzhou, China, ≥99.7%), 3,3′,4,4′-biphenyltetracarboxylic dianhydride (BPDA) (Shanghai Guchang New Chemical Materials Co., Ltd., Changzhou, China, ≥99.5%), pyromellitic dianhydride (PMDA) (Shanghai Guchang New Chemical Materials Co., Ltd., Changzhou, China, ≥99.8%), 4,4′-oxydiphthalic anhydride (ODPA) (Changzhou Sunlight Pharmaceutical Co., Ltd., Changzhou, China, ≥99.5%), 4,4‘-oxydianiline (ODA) (Changzhou Sunlight Pharmaceutical Co., Ltd., Changzhou, China, ≥99.8%), 1,1,3,3-tetramethyl-1,3-bis(3-aminopropyl)-disiloxane (TBDS) (Nanjing SISIB Silcones Co., Ltd., Changzhou, China, ≥97.3%), acetic anhydride (AA) (Sigma-Aldrich, Saint Louis, MO, USA, ≥99.5%), pyridine (Py) (Daejung Chemicals & Materials, Siheung-si, Republic of Korea, ≥99.5%), dimethyl sulfoxide (DMSO) (Daejung Chemicals & Materials, Siheung-si, Republic of Korea, ≥99.8%), *n*-hexane (Daejung Chemicals & Materials, Siheung-si, Republic of Korea, ≥98.5%), methyl alcohol (Daejung Chemicals & Materials, Siheung-si, Republic of Korea, ≥99.5%), toluene (Daejung Chemicals & Materials, Siheung-si, Republic of Korea, ≥99.8%), *p*-xylene (Daejung Chemicals & Materials, Siheung-si, Republic of Korea, ≥99.0%), tetrahydrofuran (THF) (anhydrous, Daejung Chemicals & Materials, Siheung-si, Republic of Korea, ≥99.8%), acetone (Daejung Chemicals & Materials, Siheung-si, Republic of Korea, ≥99.8%), chloroform (CHCl_3_) (Daejung Chemicals & Materials, Siheung-si, Republic of Korea, ≥99.8%), chloroform-d (Sigma-Aldrich, Saint Louis, MO, USA, ≥99.8%), *N*,*N*-dimethylacetamide (DMAc) (Alfa Aesar, Haverhill, MA, USA, ≥99.8%), and *N*,*N’*-dimethylformamide (DMF) (Alfa Aesar, Haverhill, MA, USA, ≥99.8%) were used as received. Polycarbonate (PC) and polyethersulfone (PES) were purchased from CS Hyde (Lake Villa, IL, USA).

### 2.2. Synthesis of Self-Healable PIs

**Figure 1:**
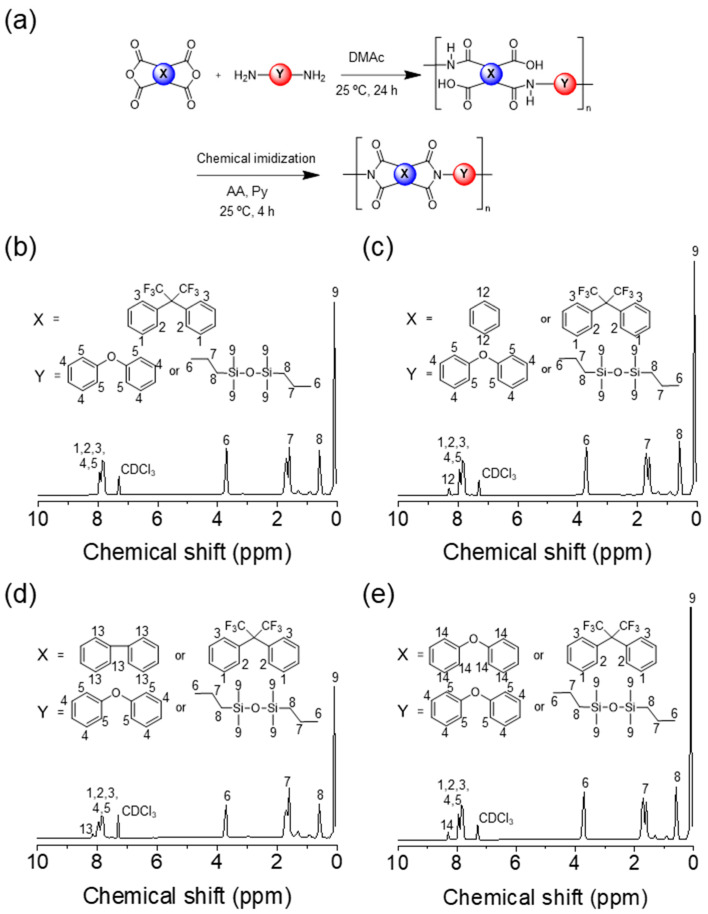
(**a**) Synthetic routes for self-healable PIs. Chemical structures and ^1^H-NMR spectra of (**b**) 6F, (**c**) P20, (**d**) B20, and (**e**) O20.

Figure 1 and Table 1 demonstrate the chemical structures and compositions of the self-healable PIs (6F, P20, B20, and O20) in this study. For the synthesis of 6F, 6FDA (19.966 g, 44.95 mmol), TBDS (11.00 g, 45.35 mmol), ODA (0.009 g 0.05 mmol), and DMF (72.275 g) were added to a double-jacketed reaction flask purged with nitrogen gas. The solution with a solid content of 30 wt% was mechanically stirred using an anchor-type impeller at 25 °C for 24 h to prepare the precursor of 6F. AA (18.447 g, 180.69 mmol) and Py (14.293 g, 180.69 mmol) were added to the solution for chemical imidization, and the resulting solution was stirred at 25 °C to prepare the 6F solution. *M*_n_ = 21,600 g/mol; *M*_w_ = 42,300 g/mol; PDI = 1.96. For the synthesis of P20, PMDA (1.961 g, 8.99 mmol), 6FDA (15.973 g, 35.96 mmol), TBDS (11.00 g, 45.35 mmol), ODA (0.009 g 0.05 mmol), and DMF (67.533 g) were added to a double-jacketed reaction flask purged with nitrogen gas. The solution with a solid content of 30 wt% was mechanically stirred using an anchor-type impeller at 25 °C for 24 h to prepare the precursor of P20. AA (18.447 g, 180.69 mmol) and Py (14.293 g, 180.69 mmol) were added to the solution for chemical imidization, and the resulting solution was stirred at 25 °C to prepare the P20 solution. *M*_n_ = 28,600 g/mol; *M*_w_ = 52,300 g/mol; PDI = 1.83. For the synthesis of B20, BPDA (2.645 g, 8.99 mmol), 6FDA (15.973 g, 35.96 mmol), TBDS (11.00 g, 45.35 mmol), ODA (0.009 g 0.05 mmol), and DMF (67.533 g) were added to a double-jacketed reaction flask purged with nitrogen gas. The solution with a solid content of 30 wt% was mechanically stirred using an anchor-type impeller at 25 °C for 24 h to prepare the precursor of B20. AA (18.447 g, 180.69 mmol) and Py (14.293 g, 180.69 mmol) were added to the solution for chemical imidization, and the resulting solution was stirred at 25 °C to prepare the B20 solution. *M*_n_ = 31,100 g/mol; *M*_w_ = 57,100 g/mol; PDI = 1.84. For the synthesis of O20, ODPA (2.789 g, 8.99 mmol), 6FDA (15.973 g, 35.96 mmol), TBDS (11.00 g, 45.35 mmol), ODA (0.009 g 0.05 mmol), and DMF (67.533 g) were added to a double-jacketed reaction flask purged with nitrogen gas. The solution with a solid content of 30 wt% was mechanically stirred using an anchor-type impeller at 25 °C for 24 h to prepare the precursor of O20. AA (18.447 g, 180.69 mmol) and Py (14.293 g, 180.69 mmol) were added to the solution for chemical imidization, and the resulting solution was stirred at 25 °C to prepare the O20 solution. *M*_n_ = 26,500 g/mol; *M*_w_ = 51,500 g/mol; PDI = 1.95. For the purification of the samples, each solution was precipitated into distilled water, and the precipitates were filtered and further purified by solvent exchange with ethanol two times. The final filtered self-healable PI powders were dried under a vacuum. For the comparison of optical properties, additional self-healable PIs (PX, BX, and OX; X = 10 and 30) were synthesized using the same preparation methods for 6F, P20, B20, and O20 (see Table S1 in the Supplementary Materials for the samples’ designations and chemical compositions).

### 2.3. Preparation of Self-Healable PCMs

A flow-coating technique was employed to prepare the self-healable PCMs in this study. The purified self-healable PI powders were redissolved in DMF to prepare the casting solutions with a solid content of 20 wt%. Each solution was homogeneously mixed and degassed using a paste mixer (PDM-300, Dae-Wha Tech., Yongin-si, Republic of Korea). The resulting solution was coated onto a glass substrate using a film applicator and thermally treated on a hot plate (60 °C for 30 min and 170 °C for 20 min) to prepare the self-healable PCMs (6F, P20, B20, and O20). For the comparison of optical properties, additional self-healable PCMs (PX, BX, and OX; X = 10 and 30) were prepared using the same preparation methods for 6F, P20, B20, and O20.

### 2.4. Characterization

The structural characterization of the samples was performed using a proton nuclear magnetic resonance (^1^H NMR) spectrometer (AVANCE III 300, Bruker, Billerica, MA, USA) with CDCl_3_ as an NMR solvent. The molecular weight characteristics of polymers were measured by gel permeation chromatography (GPC, Waters GPC system, Waters, Milford, MA, USA) with a refractive index detector using THF eluent with columns calibrated against the standard polystyrene samples. The thermal properties were investigated using thermal gravimetric analysis (TGA, Pyris 1 TGA, Perkin Elmer, Waltham, MA, USA) at a heating rate of 10 °C/min under a nitrogen atmosphere and differential scanning calorimetry (DSC, DSC 8500, Perkin Elmer, Waltham, MA, USA) at a heating rate of 10 °C/min under a nitrogen atmosphere. The surface scratches were created using a hardness test pencil (Model 318S, ERICHSEN, Hermer, Germany) with an ISO standard tip (1 mm) and a loading force of 3 N. The self-healing properties were examined using an optical microscope (HT004, Himax-tech, Seoul, Republic of Korea). The scratch depth was measured using an alpha stepper (Alpha-Step IQ, KLA-Tencor Corporation, Milpitas, CA, USA). The self-healing efficiency was calculated as follows:

(1) $$Healing\;Efficiency\left(\%,HE\right)=\frac{S-S_{H}}{S}\times 100$$

where *S* and *S*_H_ are the depths of the scratches before and after self-healing, respectively. The mechanical surface properties were investigated using a nanoindentation tester (Hysitron TI950 Triboindenter, Bruker, Billerica, MA, USA) with a conical tip of radius 20 μm, a linear loading rate of 6 mN/min from 0 to 1.0 μN, and a scratching velocity of 2 μm/min. The optical properties were obtained by spectrophotometer (CM-3600d, Konica Minolta, Tokyo, Japan). The weatherability test was conducted using a UV irradiator (CT-UV-Tunder (UV-B lamp), Dongseo Science Co., Ltd., Dangjin-si, Republic of Korea). The difference in yellow index (ΔYI) before and after exposure to UV light was calculated as follows:

$$\Delta YI={YI}_{h}-{YI}_{i}$$

where YI_i_ is the initial yellow index value and YI_h_ is the yellow index value after exposure to UV light.

## 3. Results and Discussion

### 3.1. Synthesis and Solubility

Figure 1 illustrates the synthetic routes for self-healable PIs (6F, P20, B20, and O20) in this study, and Table 1 lists their chemical compositions. It is noted that while all the samples contain the same diamine compositions of TBDS and ODA, they have different dianhydride compositions: 6F consists of 6FDA (100 in molar ratio), P20 comprises 6FDA (80 in molar ratio) and PMDA (20 in molar ratio) with a phenyl ring, B20 contains 6FDA (80 in molar ratio) and BPDA (20 in molar ratio) with a biphenyl ring, and O20 is composed of 6FDA (80 in molar ratio) and ODPA (20 in molar ratio) with two phenyl rings connected by an ether linkage. It is noted that the molar ratio of dianhydrides to diamines was adjusted to be 1:1.01 for all samples. When excess dianhydride monomers are used to synthesize PIs, the resulting PI chain terminates with dianhydride groups. It is noted that dianhydride groups, which are strong electrophiles, can readily react with environmental chemicals, including water molecules, leading to chemical transformation [53,54]. In addition, the presence of dianhydride groups at both ends of the PI chain can induce crosslinking reactions, disturbing imide formation [53,54,58]. Furthermore, in terms of controlling the molecular weight of PIs, the strategy using excess diamine monomers offers the advantage of facilitating easy processability [59,60]. Therefore, similar to most conventional PI production processes, excess molar ratios of diamine monomers were employed in this study. The structures of the synthesized PIs were investigated using ^1^H-NMR spectroscopy, as shown in Figure 1b–d. The ^1^H-NMR spectra exhibited the peaks corresponding to the protons in 6FDA and ODA (7.7–8.0 ppm) and TBDS (3.7–3.9, 1.6–1.9, 0.5–0.7, and 0.1–0.3 ppm). In addition, the ^1^H-NMR spectra of P20, B20, and O20 showed the peaks associated with the aromatic protons of PMDA (8.3 ppm), BPDA (8.1 ppm), and ODPA (8.2 ppm), respectively. The high electron density of Si contributes to the formation of an electron-shielding effect on adjacent protons [61]. As a result, the protons (9) in TBDS that were closest to Si exhibited the most significant electron-shielding effect, resulting in peaks observed at 0.1 ppm. It is noted that as the distance between Si and protons increased, the electron shielding effect diminished, causing the associated peaks to shift to downfield positions. Therefore, the peaks corresponding to protons (8), (7), and (6) appeared at 0.5 ppm, 1.6–1.8 ppm, and 3.8 ppm, respectively.

PIs are known for exceptional mechanical and thermal properties owing to CTC interactions [53,54]. It is noted that CTC interactions typically occur between aromatic structures with rich π-electrons. However, non-aromatic structures with a substantial electron density can also participate in the CTC formation through electrostatic interactions. The proton peaks of TBDS appear in the range of 0.1–0.3 ppm because of an electron-shielding effect [62,63]. This indicates that the silicon moieties in TBDS possess abundant electrons. As a result, despite the absence of aromatic structures in TBDS, it can function as an electron donator for the CTC formation.

The solubility measurements for the synthesized self-healable PIs were conducted using various common organic solvents (see Table S2 in the Supplementary Materials for the summary of solubility measurement results). PIs are known to be insoluble in most organic solvents due to the dense interchain packing resulting from strong CTC interactions [53,54], which can be a severe challenge in solution coating processes. Interestingly, in contrast to conventional PIs, 6F, P20, B20, and O20 exhibited excellent solubility exceeding 50% in common organic solvents. These results are attributed to the flexible silane moieties within the self-healable PI backbone. Those flexible groups effectively disrupt the dense interchain packing of the self-healable PIs, enhancing their chain mobility and solubility [64,65]. Consequently, the significantly increased solubility of the self-healable PIs prepared in this study holds promise for widespread applications in general solution coating processes.

### 3.2. Thermal and Mechanical Surface Properties

**Figure 2:**
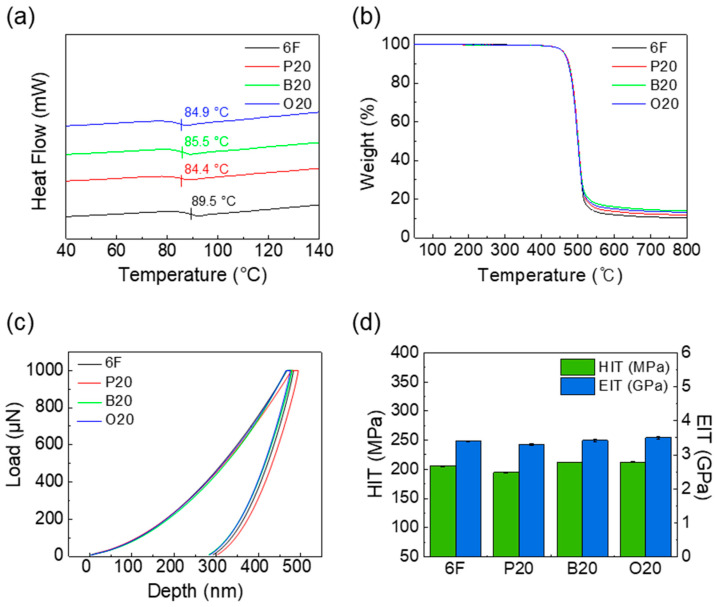
(**a**) DSC thermograms and (**b**) TGA curves of 6F, P20, B20, and O20. (**c**) Loading–unloading curves and (**d**) nanoindentation hardness (HIT) (green) and nanoindentation modulus (EIT) (sky blue) of 6F, P20, B20, and O20. All data points were obtained by averaging four measurements after excluding the highest and lowest values.

The thermal properties of the samples were examined using DSC and TGA, as shown in Figure 2a,b and Table 1. The *T*_g_s of 6F, P20, B20, and O20 were below 90 °C, which is significantly lower than those of conventional PIs. PIs typically exhibit high *T*_g_ due to the densely packed rigid chains formed by CTC interactions between electron donors and acceptors. However, a silane-based flexible spacer in TBDS led to a significant enhancement in the chain mobility of the self-healable PIs compared with traditional rigid PIs. Consequently, incorporating TBDS reduces the *T*_g_ of the PI chain.

The residual solvents and thermal decomposition temperatures (*T*_d_s) of the samples were measured, and the results are shown in Figure 2b and Table 1. The TGA curves indicated negligible weight losses within the temperature range of 50 to 200 °C. Considering the solvent (DMF) used in this study has a boiling point of 153 °C, there were no residual solvents after the thermal treatment. Introducing flexible moieties into the PI backbone improves chain flexibility and disrupts chain packing, lowering *T*_g_. However, the reduced *T*_g_ often negatively affects the thermal stability of polymers [66]. Remarkably, despite the low *T*_g_ values observed in all the samples, they exhibited a 1 wt% loss thermal decomposition temperature at 420 °C, indicating exceptional thermal properties (see Table S3 in the Supplementary Materials for the summary of various thermal degradation temperatures of self-healable PIs).

Conventional PIs are known for their high thermal stability, with thermal degradation temperatures typically observed at around 420 °C. This stability arises from aromatic monomers in the conventional PI chain. Previous studies indicate that introducing linear aliphatic monomers into PIs generally increases chain flexibility, resulting in reduced chain packing density and decreased thermal stability [65]. Because TBDS also has a linear aliphatic structure, it might be expected that TBDS-based PIs would exhibit poor thermal stability. However, self-healable PIs synthesized in this study demonstrated good thermal stability comparable to conventional PIs. This phenomenon can be attributed to the presence of silane groups in TBDS. The silane groups in TBDS possess high electron density, which can effectively induce CTC formation. In addition, the Si-O bonds in TBDS are reported to be highly thermally stable and exhibit a higher bond dissociation energy of 110 kcal/mole than C-O (85.5 kcal/mole), C-C (82.6 kcal/mole), and Si-C (72.0 kcal/mole) bonds [63]. Therefore, unlike general PIs with linear aliphatic monomers, self-healable PIs containing TBDS can maintain excellent thermal stability.

The mechanical surface properties of PCMs are crucial to ensure the surface hardness and durability of next-generation electronics and mobility. The mechanical surface properties of the samples were measured using a nanoindentation tester and compared with those of representative engineering plastics such as polycarbonate (PC) and polyethersulfone (PES). As shown in Figure 2c,d and Table 1, all the samples exhibited similar surface hardness and modulus values, suggesting that PMDA, BPDA, and ODPA did not substantially influence the chain packing in CTC. While the *T*_g_s of the samples in this study are lower than those of PC and PET [67], their mechanical surface properties are comparable with those of PC and higher than those of PES (see Figure S1 in the Supplementary Materials for the mechanical surface properties of PC and PES).

Introducing ODA with a biphenyl structure into the PI chain can enhance its rigidity, significantly improving surface scratch resistance. Non-ODA 6F, which does not contain ODA moieties, was synthesized, and its mechanical surface properties were compared with those of 6F (see Figure S2 in the Supplementary Materials for the optical microscopic images of the surface scratches of non-ODA 6F and 6F). Despite the small content of ODA in 6F, it exhibited less severe surface damage than non-ODA 6F. These results demonstrate that the enhanced chain rigidity of the PI chain leads to increased surface hardness. However, because ODA has a relatively rigid structure compared to TBDS, increasing the ODA content in the PI chain raises *T*_g_. The increased *T*_g_ restricts chain mobility and requires high temperatures to trigger self-healing. Therefore, the amount of ODA in 6F was optimized to a minimum level to improve the final mechanical surface properties while ensuring adequate chain mobility at moderate temperatures.

It is noted that conventional self-healable materials typically exhibit a trade-off relationship between their self-healing and mechanical properties [46,47]. Therefore, these results highlight that the self-healable PIs synthesized in this study effectively overcome the trade-off limitation in the traditional self-healing materials.

### 3.3. Self-Healing Properties

**Figure 3:**
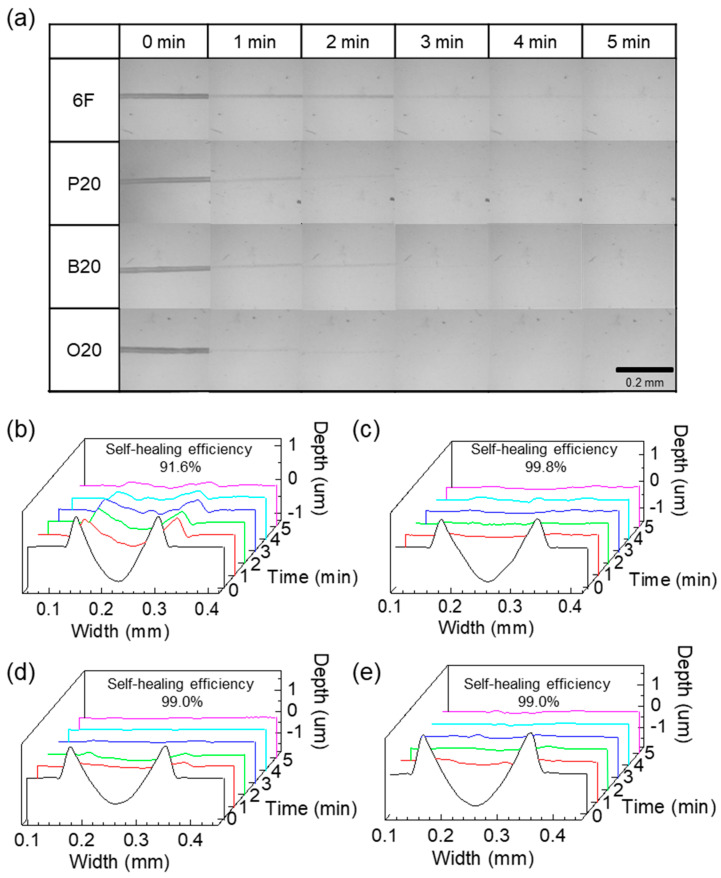
(**a**) Optical microscopic images of 6F, P20, B20, and O20 before and after self-healing. Depth profiles of (**b**) 6F, (**c**) P20, (**d**) B20, and (**e**) O20 before (0 min: black) and after (1 min: red; 2 min: green; 3 min: blue; 4 min: cyan; and 5 min: magenta) the self-healing process.

A self-healing experiment was conducted using a single-scratch technique, and the results are shown in Figure 3. A pencil-type scratch tester with a loading force of 3 N was used to generate the scratches. Previous research has reported that the thermally induced chain mobility of polymers significantly affects the self-healing process [37]. Therefore, a consistent self-healing condition (*T*_g_ + 10 °C for 5 min) was applied to each sample. Figure 3a shows the microscope images of 6F, P20, B20, and O20 before and after self-healing. While a trace of the scratch on the surface of 6F remained after the healing process, the scratches on the surface of P20, B20, and O20 disappeared within a few minutes. A depth profile was employed to quantitatively analyze the self-healing efficiency of the samples. The depths of the scratches as a function of self-healing time were measured by an alpha stepper, and the results were used to calculate the self-healing efficiencies.

As shown in Figure 3b–d, all samples exhibited rapid self-healing performance and achieved healing efficiency of over 90% within 5 min. It is noted that CTC interactions are one of the strong physical interchain interactions [53,54], and they can serve as an effective driving force for enhancing self-healing performance. Because TBDS has a silane-based flexible aliphatic spacer and abundant electrons, it can promote chain mobility and induce CTC formation, leading to high self-healing efficiency. It is noted that P20 (99.8%), B20 (99.0%), and O20 (99.0%) showed better self-healing performance than 6F (91.6%). This is attributed to the differences in the chemical structures of the self-healable PIs. In contrast to P20, B20, and O20, the dianhydride monomers used to synthesize 6F consist entirely of 6FDA (100 dianhydride mol%). The presence of two bulky CF_3_ groups in 6FDA disrupts the CTC formation in 6F by increasing the distance between PI chains. Because P20, B20, and O20 have more planar aromatic rings with π-electrons than 6F, they can facilitate stronger CTC interactions than 6F. As a result, P20, B20, and O20 demonstrated superior self-healing capabilities compared to 6F. A detailed explanation of the mechanism underlying the improved self-healing properties will be discussed in the Proposed Mechanism section.

### 3.4. Optical Properties and Weatherability

**Figure 4:**
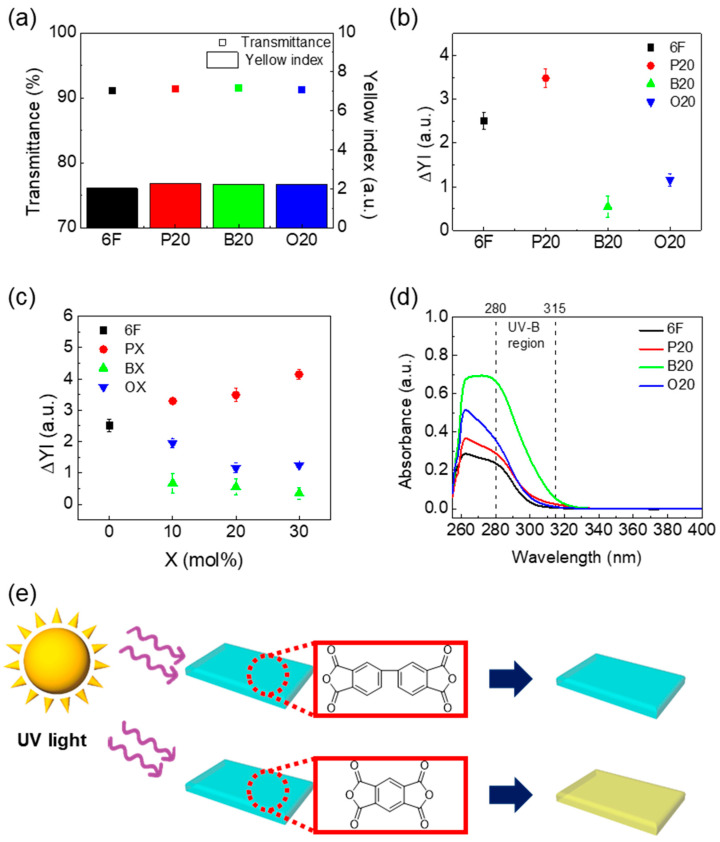
(**a**) Optical properties of 6F, P20, B20, and O20. All data points were obtained by averaging three measurements. (**b**) ΔYI after exposure to UV light for 72 h and (**c**) ΔYI results of 6F, PX, BX, and OX (X = 10, 20, and 30) after exposure to UV light for 72 h. All data points were obtained by averaging three measurements. (**d**) UV-Vis spectra of 6F, P20, B20, and O20. (**e**) Schematic illustration of UV degradation process of B20 (top) and P20 (bottom).

The optical properties were measured using a spectrophotometer, as shown in Figure 4 and Table 1. It is well known that the CTC interactions are responsible for yellowing in PIs [53,54,68]. The bulky CF_3_ groups in 6FDA reduce the chain packing density within PI structures by the steric hindrance effect, leading to the decoloration of PIs. As a result, all the samples demonstrated excellent optical properties, including transmittance above 90% and a low yellow index (YI) close to 2.0. The YI of 6F was slightly lower than those of other samples because 6F has more bulky 6FDA moieties that can disturb the CTC formation.

Figure 4b shows the difference in the YI (ΔYI) before and after UV light exposure to the samples for 72 h (see Table S4 in the Supplementary Materials for the ΔYI results for 24 and 48 h). It should be noted that B20 with a biphenyl structure showed the lowest ΔYI value after exposure to UV light. A series of self-healable PCMs (6F, PX, BX, and OX; X = 10, 20, and 30) was also prepared, and their ΔYI values for 72 h were analyzed, as shown in Figure 4c. X denotes the dianhydride molar ratio of PMDA, PBDA, and ODPA in PX, BX, and OX, respectively (see Table S1 in the Supplementary Materials for the chemical compositions of PX, BX, and OX). While the ΔYI values of PX increased in proportion to the amount of PMDA with a phenyl group, those of BX dramatically decreased with an increasing amount of BPDA with a biphenyl group.

Figure 3d shows the UV absorption spectra of 6F, P20, B20, and O20. B20 with a biphenyl group exhibited the largest UV absorption peak area compared with other samples. Previous research reported that a biphenyl structure effectively absorbs UV light owing to the conjugation length effect [69,70]. It is known that UV light generates radicals in polymers, which induces chain scission, structural degradation, and alterations in color [9,10,22]. Owing to the UV absorption capacity of BPDA, B20 demonstrates excellent weatherability against UV-induced radicals. In contrast, O20 with two phenyl rings connected by an ether linkage showed a smaller UV absorption peak area and a higher ΔYI value than B20 with a biphenyl ring. This is because an oxygen atom between phenyl rings partially disrupts the conjugated structure and diminishes UV absorption ability. P20 with a phenyl ring showed a smaller UV absorption peak area and a higher ΔYI value than B20 and O20, which results from the shortened conjugation length. 6F contains more 6FDA moieties than P20, B20, and O20. Because the high electron-withdrawing ability of the CF_3_ group in 6FDA restricts the transition of π-electrons and the formation of conjugation [71], 6F shows the smallest UV absorption peak area, as shown in Figure 4b. Consequently, it is concluded that self-healing materials involving BPDA moieties demonstrate exceptional weatherability, thus ensuring optical stability against UV exposure, as schematically shown in Figure 4e.

### 3.5. Proposed Mechanism

A mechanism for self-healing behaviors of CTC-based self-healable PCMs is proposed in this study. The CTC formation in conventional PIs is driven by electrical interactions between electron-rich aromatic diamines (serving as electron donors) and electron-deficient aromatic dianhydrides (acting as electron acceptors). In general, aliphatic and alicyclic diamines with fewer electrons are considered ineffective electron donors because of their electron-deficient nature [44]. However, the results from Figure 1b–e and Figure 3 reveal that aliphatic diamines with abundant electrons can induce effective CTC formation. Furthermore, incorporating aliphatic diamines with flexible moieties into the PI chain enhances chain mobility. As a result, self-healable PCMs with TBDS demonstrate rapid and efficient self-healing characteristics.

The self-healing efficiencies depend on the chain mobility at temperatures above *T*_g_ [28,72,73]. Therefore, elevating the self-healing temperature and extending the self-healing time are expected to augment chain mobility, improving self-healing capabilities [28,74]. It should be noted that high temperatures and prolonged exposure time at high temperatures can expedite thermal degradation [75]. Therefore, optimizing the self-healing temperature and time is essential to achieve optimal self-healing performance while mitigating significant thermal degradation.

**Scheme 1:**
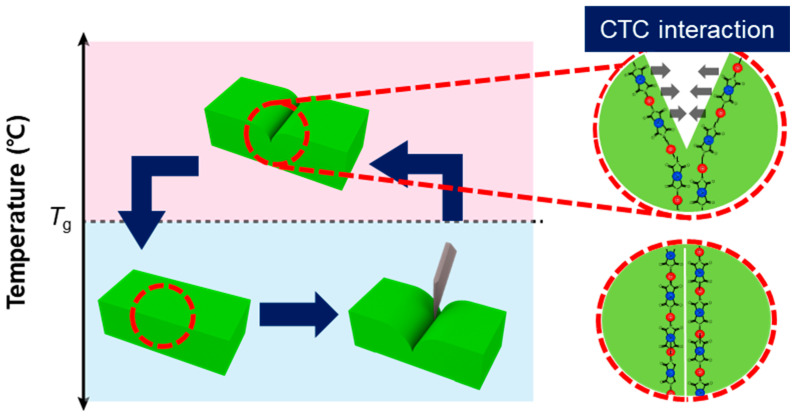
Schematic illustration of the proposed self-healing mechanism.

Based on these results, a self-healing mechanism was derived as follows. In the self-healable PCM, specific interactions between polymeric chains are crucial for effective self-healing. When scratches occur on the surfaces of self-healable PCMs, electron donors and acceptors become exposed on the surfaces of the two walls separated by the damaged area, as shown in Scheme 1. The self-healing process starts when these electron donors and acceptors on the two walls interact through the CTC formation, initiating healing from the bottom of the scratch. As the CTC reformation continues, the newly formed CTC interactions between the two walls effectively repair the damage, as illustrated in Scheme 1.

## 4. Conclusions

In this study, we developed highly self-healable PCMs with enhanced mechanical, thermal, and optical properties by utilizing CTC interactions. A flexible silane group with abundant electrons was incorporated into the self-healable PI to induce effective CTC interactions. The resulting self-healable PCMs exhibited outstanding thermal stability, rapid self-healing kinetics, and high self-healing efficiency. In addition, they showed excellent optical properties and enhanced weatherability against UV light and demonstrated superior mechanical properties to conventional engineering plastics by overcoming the limitations of conventional self-healing materials. Therefore, we expect this work to open new avenues for fabricating highly self-healable PCMs for the automotive, aerospace, munitions, healthcare, and renewable energy industries. It also can provide a route for developing new functional coating materials to protect the surface of next-generation displays and electronics, such as foldable, rollable, wearable, and stretchable devices.

## Figures and Tables

**Table 1:** Summary of compositions, thermal properties, mechanical surface properties, and optical properties of the samples in this study.

Sample Designations	Compositions							Thickness	Thermal Properties		Mechanical Surface Properties ^a^		Optical Properties ^b^		
	Dianhydrides				Diamines		(Diamines) /(Dianhydrides)		*T*_d_ ^c^	*T*_g_ ^d^	HIT ^e^	EIT ^f^	Transmittance ^g^	YI_i_ ^h^	ΔYI ^i^
	6FDA	PMDA	BPDA	ODPA	TBDS	ODA									
	(Molar Ratio)						-	(μm)	(°C)	(°C)	(MPa)	(GPa)	(%)	(a.u.)	(a.u.)
6F	100	-	-	-	99.9	0.1	1.01	2.6	422.8	89.5	205.33 ±1.24	3.39 ±0.02	91.1 ±0.10	2.02 ±0.10	2.51 ±0.20
P20	80	20	-	-			1.01	2.8	424.0	84.4	194.77 ±1.04	3.31 ±0.02	91.3 ±0.12	2.28 ±0.10	3.48 ±0.22
B20	80	-	20	-			1.01	2.0	425.5	85.5	212.23 ±0.57	3.42 ±0.05	91.6 ±0.11	2.23 ±0.08	0.55 ±0.25
O20	80	-	-	20			1.01	3.8	425.7	84.9	212.3 ±0.95	3.50 ±0.04	91.3 ±0.10	2.22 ±0.10	1.16 ±0.15

^a^ Measured by a nanoindentation tester (all data points were obtained by averaging three measurements); ^b^ measured by a spectrophotometer (all data points were obtained by averaging three measurements); ^c^ thermal decomposition temperature at 1 wt% loss; ^d^ glass transition temperature; ^e^ nanoindentation hardness; ^f^ nanoindentation modulus; ^g^ total transmittance; ^h^ initial yellow index; ^i^ ΔYI after exposure to UV light for 72 h.

## Data Availability

Data are contained within the article and supplementary materials.

## Supplementary Materials

The following supporting information can be downloaded at: https://www.mdpi.com/article/10.3390/polym15234544/s1, Figure S1: (a) Loading–unloading curves and (b) nanoindentation hardness (HIT) (green) and nanoindentation modulus (EIT) (sky blue) of PC and PES. All data points were obtained by averaging four measurements after excluding the highest and lowest values. Figure S2: The optical microscopic images of (a) non-ODA 6F (100 molar ratio of TBDS) and (b) 6F after scratches on the surface. The surface scratches were generated with a loading force of 5N. Table S1: Summary of chemical compositions of PX, BX, and OX (X: 10, 20, and 30). Table S2: Solubility measurements of self-healable PIs and a representative conventional PI (Kapton^TM^) using common organic solvents. +: soluble (over 50 wt%); -: insoluble. Table S3: Summary of thermal degradation temperatures of self-healable PIs. Table S4: Summary of ΔYI results after exposure to UV light for 24 and 48 h.
