# Supplementary material for: Highly Self-Healable Polymeric Coating Materials Based on Charge Transfer Complex Interactions with Outstanding Weatherability
Source: Polymers (Basel). 2023 Nov 27;15(23):4544. doi: 10.3390/polym15234544 (PMC10707963; doi:10.3390/polym15234544)
Supplement: Supplementary file 1 [file polymers-15-04544-s001.zip › polymers-2734574-supplementary.pdf]

Supporting Information

Highly Self-healable and Flexible Polymeric Coating Materials Based on Charge Transfer Complex Interaction with Outstanding Weatherability

Pyong Hwa Hong [1,2,†], Gyeongmin Moon [1,†,‡], Jinsil Kim [3], Kiwon Choi [4], Min Jae Ko [4,5], Ho Gyu Yoon [2,*] and Sung Woo Hong [1,6,*]

[1] Green and Sustainable Materials R&D Department, Korea Institute of Industrial Technology, 89 Yangdaegiro-gil, Ipjang-myeon, Seobuk-gu, Cheonan-si 31056, Republic of Korea
[2] Department of Materials Science and Engineering, Korea University, 145 Anam-ro, Seongbuk-gu, Seoul 02841, Republic of Korea
[3] Department of Chemical Engineering, University of Montreal, 2900 Edouard Montpetit Blvd, Montreal, QC H3T 1J4, Canada
[4] Department of Chemical Engineering, Hanyang University, 222 Wangsimni-ro, Seongdong-gu, Seoul 04763, Republic of Korea
[5] Institute of Nano Science and Technology, Hanyang University, 222 Wangsimni-ro, Seongdong-gu, Seoul 04763, Republic of Korea
[6] Convergence Research Center for Solutions to Electromagnetic Interference in Future-Mobility, Korea Institute of Science and Technology, 5 Hwarang-ro 14-gil, Seongbuk-gu, Seoul 02792, Republic of Korea
* Correspondence: hgyoon@korea.ac.kr (H.G.Y.); swhong@kitech.re.kr (S.W.H.); Tel.: +82-2-3290-3277 (H.G.Y.); +82-41-589-8675 (S.W.H.)
† These authors contributed equally to this work.
‡ Current address: MS Development Team, PI Advanced Materials, 27 Godeung 1-gil, Iwol-Myeon, Jincheon-gun 27818, Republic of Korea.

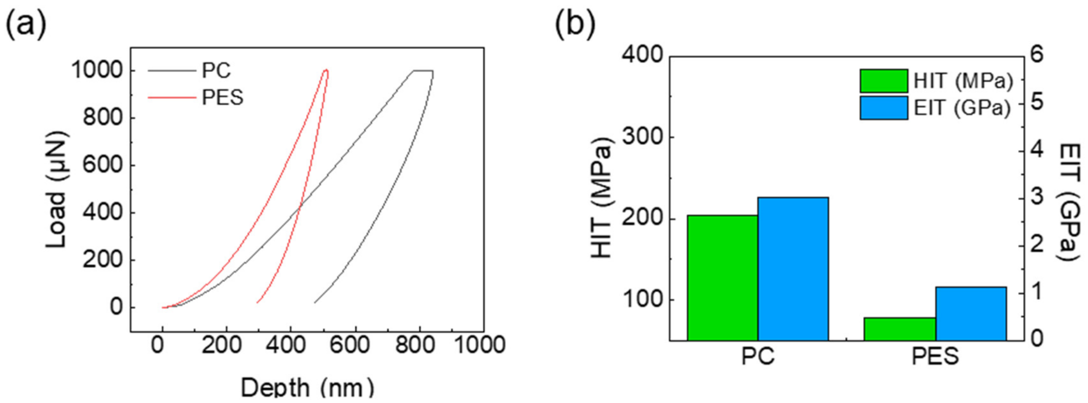

Figure S1. (a) Loading–unloading curves and (b) nanoindentation hardness (HIT) (green) and nanoindentation modulus (EIT) (sky blue) of PC and PES. All data points were obtained by averaging four measurements after excluding the highest and lowest values.

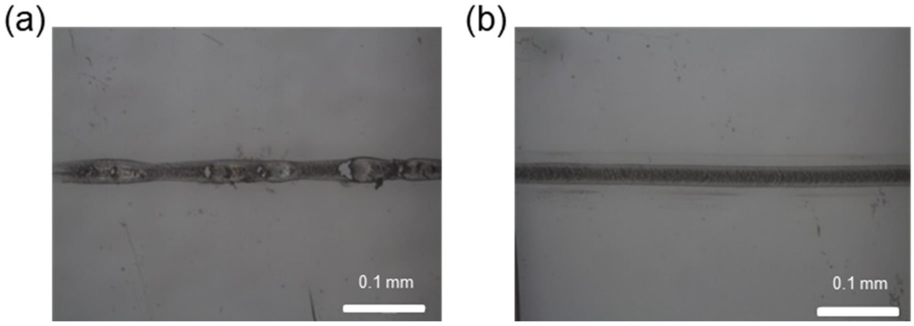

Figure S2. The optical microscopic images of (a) non-ODA 6F (100 molar ratio of TBDS) and (b) 6F after scratches on the surface. The surface scratches were generated with a loading force of 5N.

Table S1. Summary of chemical compositions of PX, BX, and OX (X: 10, 20, and 30).

Sample designations	Compositions						Thickness
	Dianhydrides				Diamines		
	6FDA	PMDA	BPDA	ODPA	TBDS	ODA	
	[molar ratio]						[μm]
P10	90	10	-	-			3.2
P20	80	20					2.8
P30	70	30					7.3
B10	90	-	10	-			1.9
B20	80		20		99.9	0.1	2.0
B30	70		30				4.4
O10	90	-	-	10			3.7
O20	80			20			3.8
O30	70			30			4.3

Table S2. Solubility measurements of self-healable PIs and a representative conventional PI (Kapton™) using common organic solvents. +: soluble (over 50 wt%); -: insoluble.

Solvents	Samples				
	Kapton™	6F	P20	B20	O20
Water	-	-	-	-	-
n-Hexane	-	-	-	-	-
Methyl alcohol	-	-	-	-	-
DMSO	-	-	-	-	-
Acetone	-	+	+	+	+
DMF	-	+	+	+	+
DMAc	-	+	+	+	+
THF	-	+	+	+	+
$CHCl_3$	-	+	+	+	+
p-Xylene	-	+	+	+	+
Toluene	-	+	+	+	+

Table S3. Summary of thermal degradation temperatures of self-healable PIs.

Samples designations	Thermal degradation temperatures			
	$T_{d,1}$[a]	$T_{d,10}$[b]	$T_{d,50}$[c]	$T_{d,max}$[d]
	[°C]			
6F	422.8	474.3	500.3	>800
P20	424.0	475.2	500.8	>800
B20	425.5	474.2	500.2	>800
O20	425.7	473.5	499.7	>800

[a]Thermal decomposition temperature at 1 wt% loss; [b]thermal decomposition temperature at 10 wt% loss; [c]thermal decomposition temperature at 50 wt% loss; [d]thermal decomposition temperature at max loss.

Table S4. Summary of ΔYI results after exposure to UV light for 24 and 48 h.

Sample designations	Optical properties[a]	
	ΔYI[b]	
	24 h	48 h
	[a.u.]	
6F	0.78±0.15	1.75±0.23
P20	2.06±0.21	2.84±0.22
B20	0.05±0.05	0.28±0.08
O20	0.13±0.06	0.70±0.12

[a]Measured by spectrophotometer (all data points were obtained by averaging three measurements); [b]YI_h-YI_i (YI_h: YI value after exposure to UV light; YI_i: initial YI value).
